# “Please listen to me”: A cross-sectional study of experiences of seniors and their caregivers making housing decisions

**DOI:** 10.1371/journal.pone.0202975

**Published:** 2018-08-30

**Authors:** Rhéda Adekpedjou, Dawn Stacey, Nathalie Brière, Adriana Freitas, Mirjam M. Garvelink, Stéphane Turcotte, Matthew Menear, Henriette Bourassa, Kimberley Fraser, Pierre J. Durand, Serge Dumont, Lise Roy, France Légaré

**Affiliations:** 1 Centre de recherche sur les soins et les services de première ligne de l'Université Laval, Université Laval, Quebec City, Canada; 2 Ottawa Hospital Research Institute and Faculty of Health Sciences, University of Ottawa, Ottawa, Canada; 3 Centre intégré universitaire en santé et services sociaux de la Capitale-Nationale, Université Laval, Quebec City, Canada; 4 Centre intégré de santé et de services sociaux de Chaudière-Appalaches, Sainte-Marie, Canada; 5 Faculty of Nursing, University of Alberta, Edmonton, Canada; 6 Faculty of Medicine, Université Laval, Quebec City, Canada; 7 Faculty of Social Sciences, Université Laval, Quebec City, Canada; 8 Department of Family Medicine and Emergency Medicine, Université Laval, Quebec City, Canada; University of Indianapolis, UNITED STATES

## Abstract

**Background:**

Little is known about the decision-making experiences of seniors and informal caregivers facing decisions about seniors’ housing decisions when objective decision making measures are used.

**Objectives:**

To report on seniors’ and caregivers’ experiences of housing decisions.

**Design:**

A cross-sectional study with a quantitative approach supplemented by qualitative data.

**Setting:**

Sixteen health jurisdictions providing home care services, Quebec province, Canada.

**Participants:**

Two separate samples of seniors aged ≥ 65 years and informal caregivers of cognitively impaired seniors who had made a decision about housing.

**Measurements:**

Information on preferred choice and actual choice about housing, role assumed in the decision, decisional conflict and decision regret was obtained through closed-ended questionnaires. Research assistants paraphrased participants’ narratives about their decision-making experiences and made other observations in standardized logbooks.

**Results:**

Thirty-one seniors (median age: 85.5 years) and 48 caregivers (median age: 65.1 years) were recruited. Both seniors and caregivers preferred that the senior stay at home (64.5% and 71.7% respectively). Staying home was the actual choice for only 32.2% of participating seniors and 36.2% of the seniors cared for by the participating caregivers. Overall, 93% seniors and 71% caregivers reported taking an active or collaborative role in the decision-making process. The median decisional conflict score was 23/100 for seniors and 30/100 for caregivers. The median decision regret score was the same for both (10/100). Qualitative analysis revealed that the housing decision was influenced by factors such as seniors’ health and safety concerns and caregivers’ burden of care. Some caregivers felt sad and guilty when the decision did not match the senior’s preference.

**Conclusion:**

The actual housing decision made for seniors frequently did not match their preferred housing option. Advanced care planning regarding housing and better decision support are needed for these difficult decisions.

## Introduction

Action must be taken to assure that seniors can age in desirable, affordable, and appropriate homes, communities and care environments; particularly given that life expectancy is projected to reach new heights and the baby-boom generation is reaching 65 years and older [1, 2]. As suggested by two recent studies about Canadians’ geographical mobility [3, 4], the majority of very old people (85+) are still living at home, for the most part because they want to [5–7]. However, with aging, seniors are at risk for chronic illness and functional limitations that eventually lead to difficulties in performing activities of daily living [8, 9]. Help from family, friends or home care services are thus crucial for limiting the consequences of this loss of autonomy [10]. One in four of Canadians aged 65 or older report receiving care through formal home care services (such as the local home care team) or informal caregivers (e.g., family/friends) [11]. These findings are similar to those reported in the United States where 23% have at least two disabilities requiring care [12]. When seniors need more care, they are often faced with the difficult decision about whether to stay at home or move to another location, such as private or public long-term care facilities, assisted living facilities, etc. [8].

Seniors having more control over the decision to move is positively associated with longer life expectancy, health, morale, life satisfaction, and overall adjustment [13]. In addition, where people live is closely related to neighborhood, and there is some evidence that individuals with negative perceptions of their neighborhood have lower levels of well-being [14]. Moreover, unmet residential mobility preferences are associated with poor subsequent mental health [15]. However, for people with cognitive disorders such as dementia, decisions are too often made only after profound changes in verbal communication preclude their ability to express their wishes [16]. In this regard, caregivers can play a significant role as surrogate decision makers [17]. However, where seniors reside can impact caregivers’ life in many ways: a) they have to balance caregiving, work responsibilities and other family obligations; b) when disabled seniors live with them, they may need to make physical and social changes to their own home to accommodate their needs; c) increased presence of healthcare professionals, alarms and other technologies may disrupt their everyday routines. Thus, it is important to assess both seniors’ and caregivers’ needs regarding decision-making about housing options. Inadequate knowledge about the options, unclear values, or undue social pressure towards one specific option may reduce the quality of the decision-making process. A decision-making process is of high quality when, during the process, clinicians and patients share the best available evidence, patients are supported to consider their options, and they can make decisions that are in line with their values and preferences. Decisions that are not well-informed or based on patients’ values and preferences have a negative impact on behavior (e.g. delays in making the choice), health outcomes, emotions (e.g., regret, blame), and may incur inappropriate use and costs of services [18, 19]. Most of the literature on seniors and informal caregivers’ housing decisions is qualitative in nature [20] and pays little attention to objective decision-making measures [21]. Qualitative data are informative but insufficient to assess the effect of a decision support intervention on the quality of the decision-making process. To fill this knowledge gap, the authors aimed to report on experiences of housing decisions among cognitively competent seniors and caregivers of cognitively impaired seniors using both quantitative and qualitative data.

## Methods

### Study design

A cross-sectional study with data collected prior to a large cluster randomized trial from interprofessional (IP) home-care teams and their clients in the province of Quebec, Canada (DOLCE trial) [22]. This larger trial was to test an interprofessional approach to shared decision making in home care, and was a response to teams working in home care settings who identified the need for decision support for seniors and informal caregivers making housing decisions. The data in the present study were collected to assess comparability between the intervention and control arms of the DOLCE trial. When this data was analyzed, interesting results emerged that could contribute to a deeper understanding of seniors’ and caregivers’ experiences of decision-making about housing. This paper presents an analysis of the quantitative data, enriched by qualitative data collected alongside it, from two separate samples: a) cognitively competent seniors aged ≥ 65 years and b) informal caregivers of cognitively impaired seniors who had made a decision about housing on behalf of their loved ones (i.e. as proxy decision-makers). Ethics committee review approval was obtained from the CHU de Québec Multicenter Ethics Committee (MP-CHU-QC-14-001).

### Conceptual framework

**Fig 1:**
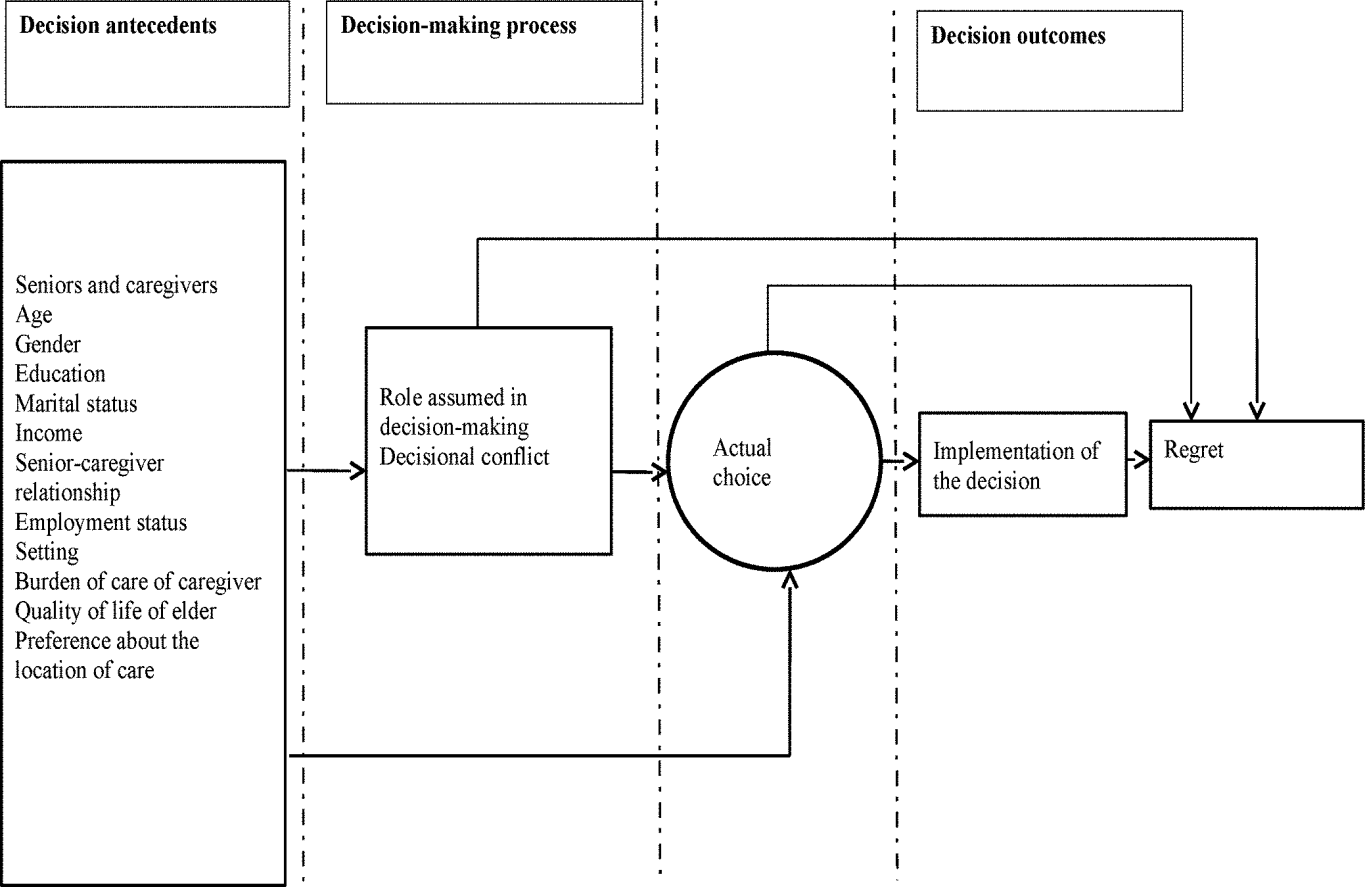
Conceptual framework of decision-making about seniors’ housing decisions (adapted from Sepucha and Mulley’s model of medical decision-making [23]).

Decision-making is a cognitive process that results in making a choice among various options (including the option of doing nothing). Decision-making models suggest that in the case of housing decisions (choices made about a living environment for seniors who need more care), three broad categories of factors are involved: decision antecedents, the decision-making process, and decision outcomes (Fig 1) [23]. Decision antecedents are the characteristics of seniors, informal caregivers, healthcare providers or organization of care that may influence or facilitate the decision-making process and their housing choice. The decision-making process focuses on the interaction between the senior, the caregiver and healthcare provider(s) (level of involvement, use of decision support tools) and the amount and type of deliberation. Decision outcomes are consequences of the choice, such as implementation of the chosen option, regret and health outcomes. This framework guided quantitative and qualitative data analysis in this study.

### Setting and participants

The setting was Health and Social Service Centres (HSSC) in the eastern part of the Province of Quebec (Canada). HSSC (known in Quebec as Centres de santé et de services sociaux) combine local community service centers, long-term care facilities and, in most cases, a hospital. The HSSC is responsible for providing the local population with accessibility, continuity and quality of care [22]. Eligibility criteria for HSSCs were that: a) they served a geographical area with a population of over 10,000 inhabitants; and that b) their distance from Quebec City (location of research team) was less than 500 km [24]. Sixteen regional HSSC were enrolled. Eleven were in rural areas and five in urban/semi-urban areas. Within each HSSC, interprofessional home-care teams providing care and decision support to seniors facing housing decisions were recruited. An interprofessional team is defined as a minimum of two healthcare providers from different professions who collaborate to provide integrated and cohesive client care [25, 26]. Eligible interprofessional home-care teams a) were involved in caring for seniors with loss of autonomy, and b) practised in one of the HSSCs selected to participate in the DOLCE trial. At study entry, 271 healthcare providers who were members of the interprofessional teams completed a questionnaire collecting information about their sociodemographic characteristics and their intention to engage in an interprofessional approach to shared decision making. Their median age was 36.1 years. Most were female (90.4%) and their median years of experience in home care was six. More details about the healthcare professional sample will be reported in a separate paper. Eligible seniors had made a decision about whether to stay at home or move to a long-term care facility in the previous six months and were: i) aged ≥65 years; ii) receiving care from the interprofessional home care team; iii); iv) able to read, understand and write French or English; and v) able to give informed consent. For seniors who could not provide informed consent, the primary informal caregiver involved in the housing decision was invited to participate. Capacity to consent of the seniors was determined according to the clinical judgement of the interprofessional home-care team members. Based on patient files, eligible participants were identified by a health professional from each HSSC, and then contacted consecutively by a research assistant (RA) until the targeted sample size of five participants per HSSC was reached. Before taking part in the study, all participants (interprofessional home-care team members, seniors and caregivers) read and signed written informed consent forms. Each participant was free to withdraw from the project at any time, by simple verbal notice, without giving the reasons of that decision.

### Data collection

The RA collected data using two closed-ended questionnaires, one for eligible seniors and the other for caregivers, to be completed at their residence with an RA. Data was collected prior to the random allocation of the enrolled HSSC for the DOLCE trial [22]. Data collection among seniors included sociodemographic characteristics, health-related quality of life (HR-QoL), role assumed in the decision-making, decisional conflict, preferred housing option, the actual choice made, and decision regret. Data collection for caregivers included the same items with the addition of items about burden of care and about the housing choice they would prefer for their loved one as well as what they considered the preference of their loved one to be.

Sixteen RAs were trained to take handwritten notes in standardized logbooks during visits with seniors and caregivers and to complete their notes within 24 hours of each meeting. RAs observed participants (noting e.g. visible signs of emotion) as they completed the study questionnaires and paraphrased participants’ narratives of their experiences volunteered orally and made other observations. Logbooks were completed for each participant in the study. Interviews were not recorded but information in each logbook was compared with that in the corresponding questionnaire to see if they were congruent. Most RAs were health professionals knowledgeable about services for seniors in their region.

### Measurements

Guided by the four main categories of the chosen conceptual framework, measures included decision antecedents (e.g., health-related quality of life, caregiver burden), decision-making process (e.g., role in decision making; decisional conflict), actual choice, and decision outcomes (e.g., decision regret).

#### Health related quality of life (HR-QoL)

Seniors’ perceived HR-QoL was assessed using the Nottingham Health Profile (NHP) questionnaire, a generic quality of life survey used to measure subjective physical, emotional and social aspects of health. One part of the survey measures six dimensions of health (physical mobility, pain, social isolation, emotional reactions, energy and sleep) and the other part consists of yes/no statements about seven areas of life that are most affected by health status [27]. Two of the six dimensions of health, namely, social isolation (five items) and emotional reactions (nine items) were assessed. For each item the scores range from 0 to 100. The higher the score, the worse is the perception of HR-QoL [27]. Test-retest correlation coefficients range from 0.77 to 0.78 for social isolation and 0.75 to 0.80 for emotional reaction [27]. The NHP differentiates successfully between seniors who do not consult general practitioners, those who are physiologically “fit” and those with chronic illnesses [28].

#### Caregivers’ burden of care

Caregivers’ burden of care was measured using the Zarit Burden Interview (ZBI). Total scores range from 0 to 88, with a score from 0 to 20 meaning little or no burden; 21 to 40 meaning mild to moderate burden; 41 to 60 meaning moderate to severe burden, and 61 to 88 meaning severe burden [29, 30]. Internal consistency of the scale is good to excellent (Cronbach alpha correlation coefficients range 0.85 to 0.93) [29, 31]. The test-retest reliability is high (intra-class correlation coefficient 0.89) [29, 31]. The ZBI score is highly correlated with the Burden Assessment Scale score (correlation coefficient = 0.73, P<0.0001) and the 28-item version of the General Health Questionnaire score (correlation coefficient = 0.62, P<0.0001), a scale that assesses psychological distress [31].

#### Role assumed in the decision-making

A modified version of the Control Preference scale (CPS) [32] was used to assess the role assumed in the decision-making reported by the senior or the caregiver [33]. The scale consists of a single question to assess the client’s perception of locus of control over the decision-making process. Response options are: A) I made the decision, B) I made the decision after seriously considering my providers’ opinions, C) my providers and I shared the responsibility for the decision-making, D) my providers made the decision after seriously considering my opinion, E) my providers made the decision [32]. A and B represent a senior- or caregiver-controlled decision-making process (active role), C represents a shared decision-making process (collaborative role), and D and E represent a provider-controlled decision-making process (passive role). The scale has moderate test-retest reliability (intra-class correlation coefficient 0.5) [34]. Agreement between self- and researcher-rated decisional roles on the CPS is good (Kendall's tau-b 0.82) [35].

#### Decisional conflict

Decisional conflict of the senior and caregiver was assessed using the Decisional Conflict Scale (DCS). The DCS measures individuals’ perceptions of uncertainty in choosing options, factors contributing to that uncertainty (such as feeling uninformed, unclear about personal values and unsupported in decision-making) and perceived effectiveness in decision-making. The DCS has 16 items, each with response statements on a 5-point Likert scale (from strongly agree (0) to strongly disagree (4)). We converted scores to a 0–100 scale: 0 meaning no decisional conflict and 100 meaning extremely high decisional conflict. Scores lower than 25 are associated with implementing the decision while scores higher than 37.5 are associated with delaying the decision or feeling unsure about acting on it. Test-retest and Cronbach alpha correlation coefficients exceed 0.78. The scale correlates to knowledge, regret and discontinuance. It discriminates between those who make and those who delay decision (effect size ranges 0.4 to 0.8) and is responsive to change (effect size ranges 0.4 to 1.2) [36].

#### Actual choice about housing

To assess the actual choice about housing, participants answered one question with five response options: stayed at home, stayed at home with home care, moved to a private care facility, moved to a public care facility, other option implemented (asked to specify). For analysis this variable was dichotomized into two categories: stayed at home or moved to another location.

#### Decision regret

We used the Decision Regret Scale to assess regret after the decision. Participants indicated whether they agreed or disagreed with the statements ‘it was the right decision’, ‘I regret the choice that was made’, ‘I would go for the same choice if I had to do it over again’, or ‘the choice did me a lot of harm’ and ‘the decision was a wise one’ by choosing among five statements ranging from strongly agree (1) to strongly disagree (5). The total scores were converted to a 0–100 scale: higher scores reflect higher decision regret. Cronbach alpha correlation coefficients ranges exceed 0.81. The scale correlates with satisfaction with the decision (r = -0.40 to -0.60), decisional conflict (r = 0.31 to 0.52) and overall rated quality of life (r = -0.25 to -0.27). It discriminates between groups who differ on feelings about the decision (negative, mixed, or positive) and between those who changed their decision and those who did not [37].

### Data analysis

The target sample size was five seniors/caregivers per HSSC for a total of 80 participants. During the development of the DOLCE trial protocol, this number was deemed enough to be able to compare HSSCs (experimental versus control) at trial entry on the basis of client profiles [38]. Descriptive statistics of the seniors’ and caregivers’ sociodemographic characteristics were computed (e.g., age, sex, marital status, employment status, education level, total family income, setting, relationship between caregiver and senior), as well as seniors’ perceived HR-QoL, caregivers’ burden of care, and seniors’ and caregivers’ decision-making experiences (participants’ housing preference, the actual choice, role assumed in decision-making, decisional conflict, decision regret). We did not compare data between seniors and caregivers, as caregivers were proxies for other seniors with cognitive impairment and these two groups represent different populations. This analysis was performed using SAS 9.4 statistical software.

Logbooks of the RA encounter with each participant were qualitatively analyzed using a hybrid deductive/inductive thematic approach [39]. With identical copies of each logbook, two authors (RA and MM) independently coded the RAs’ notes deductively by highlighting paraphrases corresponding to key ideas with markers of four different colors, each corresponding to a main category of the conceptual framework (i.e. decision antecedents, decision-making process, actual choice, decision outcomes). For the inductive analysis, an iterative three-stage process was used. At the first stage, the two authors independently analyzed 15 of the logbooks. Through discussion, they established a common coding structure and applied it to the remaining 64 logbooks. At the second stage, based on the key ideas deduced earlier from the logbook notes, each author identified sub-themes within each broad category of the conceptual framework. They then met to discuss the sub-themes and resolve disagreements. At the third stage, the two authors independently grouped each sub-theme into broader themes and met again to resolve any disagreements. At each stage, the authors discussed and further refined the classification of key ideas into sub-themes, and sub-themes into themes, which in turn gave new insight into the four categories of the conceptual framework. Given the complimentary nature of the data provided by seniors and caregivers and our intention to report results descriptively, we analyzed and reported on the data from seniors and caregivers in a combined manner.

## Results

**Fig 2:**
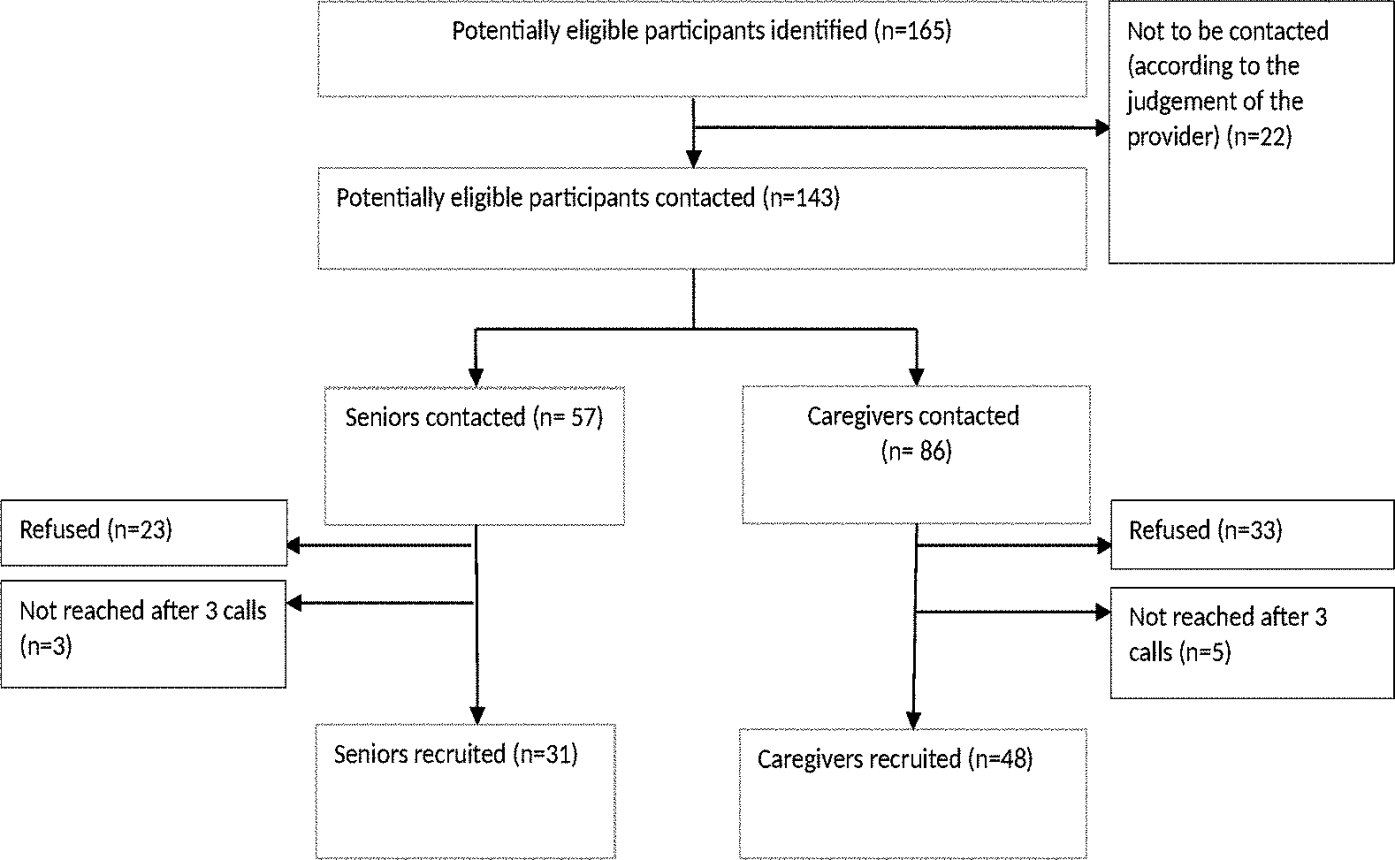
Flow chart of recruitment.

Of 143 potentially eligible individuals contacted, 31 seniors and 48 caregivers were recruited with a response rate of 55.2% (54.4% for the seniors and 55.8% for the caregivers) (Fig 2).

### Decision antecedents

Seniors were 85.5 years of median age (IQR: 78.9–89.5), were mostly female (83.9%), separated/divorced/widower (77.4%) and had completed primary education only (64.5%). Regarding seniors’ HR-QoL, medians of social isolation and emotional reaction were 22 (IQR: 0–42.1) and 11.8 (0–27.3) respectively. Caregivers were 65 years of median age (IQR: 56.4–79.2), most were female (70.8%), husband/wife/child of the senior (91.6%), completed post-secondary education (52.1%) and experienced mild to moderate burden of care (median: 32; IQR: 21–51) (Table 1).

**Table 1:** Characteristics of participants (decision antecedents).

Characteristics n (%)^a^	Seniors (n = 31)	Caregivers (n = 48)
**Age (year), median (IQR)**^**b**^	85.5 (78.9–89.5)	65.1 (56.4–79.2)
**Female**	26 (83.9)	34 (70.8)
**Marital status**		
Single	1 (3.2)	6 (12.5)
Married/Common law	6 (19.4)	30 (62.5)
Separated/Divorced	3 (9.7)	9 (18.7)
Widower	21 (67.7)	3 (6.3)
**Employment status**		
Employed	-	14 (29.2)
At home/Retired	-	34 (70.8)
**Education level**		
Primary	20 (64.5)	9 (18.7)
Secondary	4 (12.9)	12 (25.0)
Post-secondary	7 (22.6)	25 (52.1)
Other	0 (0.0)	2 (4.2)
**Total family income (CAD)**^**c**^		
<15000	4 (12.9)	7 (14.6)
15000–29999	19 (61.3)	12 (25.0)
30000–44999	0 (0.0)	8 (16.7)
45000–59999	1 (3.2)	9 (18.7)
60000 and more	1 (3.2)	5 (10.4)
No answer	6 (19.4)	7 (14.6)
**Setting**		
Urban/semi-urban	8 (25.8)	18 (37.5)
Rural	23 (74.2)	30 (62.5)
**Relationship to senior**		
Husband/wife	-	17 (35.4)
Child	-	27 (56.2)
Other family member/friend	-	4 (8.4)
**HR-QoL**^**d**^**, median (IQR)**		
Social isolation (0–100)	22.0 (0–42.1)	-
Emotional reaction (0–100)	11.8 (0–27.3)	-
**Burden of care (0–88)**^e^**, median (IQR)**	-	32 (21–51)

^a^ n: number, %: percentage (unless otherwise specified).

^b^ IQR: Interquartile range.

^c^ CAD: Canadian dollars.

^d^ Health-Related Quality of Life. The higher the score the worse is the perception of HR-QoL.

^e^ Scores between 0 to 21: little or no burden; 21 to 40: mild to moderate burden; 41 to 60: moderate to severe burden; 61 to 88: severe burden.

The preference of 64.5% of the cognitively competent seniors and 71.1% of the cognitively impaired seniors (as reported by their caregivers) was to stay at home with or without home care services (Table 2). Qualitative results corroborated these preferences of seniors and caregivers, though other family members had conflicting preferences (Table 3). For example,

*… He kept telling me about his son who tried to move them into a home*, *him and his wife*, *into a nursing home*, *saying that it would be best to sell their house*, *which he has always categorically refused to do (Spouse/partner of a senior*, *92 years)*.

**Table 2:** Preference about housing options and decision-making experiences of seniors and caregivers.

Variables n (%)^a^	Seniors (n = 31)	Caregivers (n = 48)
***Decision antecedents***		
**Preference about the housing options**^**b**^		
Stay at home	20 (64.5)	33 (71.7)
Move to another location	11 (35.5)	13 (28.3)
***Decision making process***		
**Assumed role in decision-making**		
Active role	26 (83.9)	27 (56.2)
Collaborative role	3 (9.7)	7 (14.6)
Passive role	2 (6.4)	14 (29.2)
**Decisional conflict, median score out of 100 (IQR)**^**d**^	23.4 (7.8–37.5)	30.5 (11.7–45.3)
**Decisional conflict score** **≥** **37.5 out of 100**^**e**^	8 (25.8)	16 (33.3)
***Actual choice about Housing options***^**c**^		
Stay at home	10 (32.2)	17 (36.2)
Move to another location	21 (67.8)	30 (63.8)
***Decision outcomes***		
**Decisional regret, median score out of 100 (IQR)**	10 (0–25)	10 (0–25)

^a^ n: number, %: percentage (unless otherwise specified).

^b^ Two missing values among the caregivers. In the caregivers group, preference referred to preference of cognitively impaired seniors according to the caregiver.

^c^ One missing value among the caregivers.

^d^ IQR: Interquartile range.

^e^ Four missing values among caregivers.

**Table 3:** Decision antecedents (qualitative themes that emerged from encounter logbook analysis).

Themes	Sub-themes	logbook notes^a^
**Health issue**	Cognitive disorders	*[The senior] also suffers from dementia*, *which affects her short-term memory (Daughter of a senior*, *51 years)*
	Multimorbidity	*[The senior]’s had diabetes*, *and had a stroke 15–16 years ago*, *and she also has mild Alzheimer’s (her incontinence means she’s had to wear diapers since July*, *and she has a dressing on her back which has to be changed every day)*. *She’s also had a bowel operation*, *as she had bowel cancer… And her pneumonia made it difficult for her to breathe*. *She was also taking medication because of a swollen leg (Spouse/partner of a senior*, *73 years)*
	High needs for services	*[The senior] had to take several medications and she’s incontinent*. *She has a heart problem and her blood pressure has to be monitored constantly*. *A nurse comes to their house three times a week (Spouse/partner of a senior*, *92 years)*
**Safety issues**	Falls	*Her loved one (mother) kept having falls at home*. *She had a bad fall and ended up in the hospital… (Daughter of a senior*, *51 years)*
	Dangerous behaviours	*[The senior’s] behavior became dangerous for him and for others … (Daughter of a senior*, *55 years)*
	Cleanliness	*She spent a few weeks in hospital after open heart surgery*, *so she needed a clean environment and not somewhere that was under renovation*. *(Senior*, *87 years)*
**Daily functioning**	Inability to deal with household tasks	*She recently had health problems and she doesn’t think she has the strength to look after her place any more (Senior*, *73 years)*
**Characteristics of living conditions**	Living alone vs with others	*Ms*. *X is never alone*, *there’s always someone with her*, *even at night*. *She’s well looked after by her family*. *She just lost her husband in December (Senior*, *89 years)*
	Rules for occupancy	*With Mr*. *X’s illness getting worse*, *Ms*. *X had to consider moving him somewhere else*, *because the residence where they live doesn’t accept people who are losing their autonomy (Friend of a senior*, *84 years)*
	Higher vs lower cost of living at home	*This woman … decided to leave her home and move into a private residence because her property taxes were too high (Senior*, *83 years)*
		*In addition*, *a residence for independent seniors costs much more than where she lives now (Senior*, *87 years)*
	Home adaptation	*He installed a safety gate across the outside steps (Spouse/partner of a senior*, *92 years)*
		*Ms*. *X’s present residence isn’t dangerous at all*. *There are signs posted everywhere to help her and the people who come to her place (Senior*, *89 years)*
**Caregiver burden**	Constant monitoring	*The four children went to her place (morning*, *noon and night) (Daughter of a senior*, *51 years)*
	Stress and exhaustion	*He began to get exhausted from looking after her*. *Her situation worried him a lot and it was too much for him (Spouse/partner of a senior*, *73 years)*
	Being alone in providing care	*She has to check up on her mother every day*, *she can’t rely on help from the family or from social services (Daughter of a senior*, *59 years)*
**Caregiver ability to manage care**	Caregiver ability to manage care	*He says he is perfectly capable of taking care of his wife … he makes sure she takes her medication*, *he changes her diapers*, *does the shopping*, *the laundry*, *the housework etc*. *… He has a driving licence*, *and he can stay close to home for the groceries*, *the pharmacy*, *the bank and other small shopping needs (Spouse/partner of a senior*, *92 years)*
**Previous living experiences**	Negative experiences	*Ms*. *X also had a poor opinion of long-term care facilities*. *She’s had to stay in them once in a while*, *and did not like the experience at all (Senior*, *87 years)*
**Preferences about housing options**	Elder preferences	*She would agree to move to a place that is simple*, *but well-built and insulated*. *She doesn’t need to live in a castle*. *The place has to be affordable*, *and it shouldn’t have corridors (it should be open plan)*, *and the distance between the entrance to the building and her place should be relatively short (Senior*, *87 years)*
	Caregiver preferences	*He mentions … that it’s better for her to stay at home (Spouse/partner of a senior*, *92 years)*
	Mismatching preferences (caregiver and family)	*He kept telling me about his son who tried to move them into a home*, *him and his wife*, *into a nursing home*, *saying that it would be best to sell their house*, *which he has always categorically refused to do (Spouse/partner of a senior*, *92 years)*

^a^Logbooks were completed by research assistants. A total of 79 logbooks were analyzed.

Qualitative findings also suggested that seniors’ health and safety issues, living conditions, ability to function at home, and previous living experiences influenced the decision-making process and choice of housing. The stress and burden among caregivers and their ability to manage that burden were also noted as major factors.

### Decision-making process

Most of the seniors (83.9%) and 56.2% of caregivers reported taking an active role in the decision-making. Smaller percentages of seniors and caregivers (9.7% and 14.6%, respectively) reported that the responsibility for decision-making was shared with the provider. Median decisional conflict scores (DCS) were 23.4/100 (IQR: 7.8–37.5) for the seniors. For caregivers, these scores were 30.5/100 (IQR: 11.7–45.3) (Table 2). According to the qualitative data, participants reported that the decision-making process was emotional and difficult (Table 4). The nature of this process was either *planned*, with regular assessments of the senior’s living situation and advanced planning of future housing or, more commonly, *reactive*, often triggered by hospitalizations or acute health events leaving little time to gather information on housing options. Some caregivers reported family pressures to relocate their loved one towards more institutional settings. Some caregivers were satisfied with the decision support they received from health professionals, while others were not. For example,

*The caregiver doesn’t seem to be satisfied with the services offered for supporting them in making the decision*. *Also*, *she doesn’t think the functional abilities assessment was done properly* (Daughter of a senior, 69 years)

**Table 4:** Decision making process (qualitative themes that emerged from encounter logbook analysis).

Themes	Sub-themes	logbook notes^a^
**Decision-making process experience**	Difficult decision-making process	*Ms*. *X is very emotional and tells me about what she’s been through with Mr*. *X*, *and she seems to have had a lot of trouble managing the situation (Spouse/partner of a senior*, *84 years)*
**Participants in the decision**	Family members involved in the decision	*[The senior] had a bad fall and ended up in the hospital*, *and at that point the children convinced their mother that it would make more sense for her to live in a nursing home*, *which she accepted (Daughter of a senior*, *51 years)*
**Decision supports**	Supports from health professionals	*During the decision*, *Ms*. *X was very well advised by the health professionals caring for her and her partner*. *Someone even went apartment visiting with her to evaluate what would be best for her and Mr*. *X (Friend of a senior*, *84 years)*
	Dissatisfaction with support provided	*The caregiver doesn’t seem to be satisfied with the services offered for supporting them in making the decision*. *Also*, *she doesn’t think the functional abilities assessment was done properly (Daughter of a senior*, *69 years)*
**Planned vs reactive nature of decision-making initiation**	Advanced planning of future Housing options	*He mentioned that they already had a place reserved for them in a long-term care facility when they were ready to move there (Spouse/partner of a senior*, *92 years)*
	Family regular assessments of senior's living situation	*About every month*, *she and her husband evaluate whether [her mother] can go on living with them (Daughter of a senior*, *64 years)*
	Hospitalization/acute health event triggers decision-making process	*Ms*. *X explained that after she was hospitalized in August*, *she made the decision to move into a private seniors’ residence (Senior*, *87 years)*
		*Ms*. *X and her daughter*, *Ms*. *Robert*, *considered the move after Mr*. *Robert had been hospitalized (Daughter of a senior*, *55 years)*
	No time to gather information on options	*For Mr*. *X*, *the decision to move happened so fast (less than half a day) that he hadn’t had time to find out about other options open to him (Daughter of a senior*, *55 years)*
**Pressure from family**	Pressure from family members	*He kept talking to me about his son who had tried to move them into a home*, *him and his wife*, *into a nursing home… He said that for a year*, *it was very hard for him*, *this pressure from his son*. *… Then he managed to get his son to understand that he wanted to stay in his house and told him to stop talking to him about it (Spouse/partnerof a senior*, *92 years)*

^a^Logbooks were completed by research assistants. A total of 79 logbooks were analyzed.

### Actual choice about housing option

Staying at home was the actual choice for 32.2% of seniors and 36.2% of caregivers (Table 2). Qualitative results suggested that in many instances the actual choice did not reflect their initial choice to remain at home (Table 5). For example,

*She was forced to move*, *because her landlord had started renovations … and in addition*, *she got sick at the same time … her son took this opportunity to move her into a seniors’ residence* (Senior, 87 years)

*Mr*. *X had a stroke*. *He can’t speak any more*. *His non-verbal communication is very forceful*. *He always wants her to take him home* (Spouse/partner of a senior, 80 years)

**Table 5:** Actual choice (qualitative themes that emerged from encounter logbook analysis).

Themes	Sub-themes	logbook notes^a^
**Nature of the choice**	Difficult	*The woman decided to put her house up for sale in the near future*, *and she finds that hard (Senior*, *83 years)*
	Forced	*She was forced to move*, *because her landlord had started renovations … and in addition*, *she got sick at the same time … her son took this opportunity to move her into a seniors’ residence (Senior*, *87 years)*
**Match of preferences to actual choice**	Match of preferences to actual choice	*She and her husband think they’ll stick to their decision to keep her mother at home with them for as long as possible*. *She thinks this is what her mother wants… (Daughter of a senior*, *64 years)*
	Mismatch between preferences and actual choice	*Mr*. *X had a stroke*. *He can’t speak any more*. *His non-verbal communication is very forceful*. *He always wants her to take him home (Spouse/partner of a senior*, *80 years)*

^a^Logbooks were completed by research assistants. A total of 79 logbooks were analyzed.

### Decision outcomes

Median decisional regret scores were 10/100 for both seniors and caregivers (Table 2). Other decision outcomes reported qualitatively by participants were both positive and negative (Table 6). Some were satisfied with the decision to move and with the new living arrangements, and others were satisfied with the decision to stay home because of the lower cost:

*… a residence for independent seniors costs much more than where she lives now*, *without offering her more in services or guaranteed available help 24 hours a day*, *which is what Ms*. *X would need* (Senior, 87 years)

**Table 6:** Decision outcomes (qualitative themes that emerged from encounter logbook analysis).

Themes	Sub-themes/	logbook notes^a^
**Positive outcomes**	Satisfaction with the decision to move	*… [The caregiver] was very satisfied with the decision in spite of the fact that it had been hard to make (Spouse/partner of a senior*, *79 years)*
	Satisfaction with the new living place	*Ms*. *X is very satisfied with the place where she lives now and has been happy ever since she moved (Daughter of a senior*, *55 years)*
	Lower cost of living	*In addition*, *a residence for independent seniors costs much more than where she lives now*, *without offering her more in services or guaranteed available help 24 hours a day*, *which is what Ms*. *X would need (Senior*, *87 years)*
**Negative outcomes**	Dissatisfied with the decision to move	*Ms*. *X clearly stated that she is very dissatisfied with the decision to move*. *However*, *this dissatisfaction occurred after the move (Senior*, *74 years)*
	Dissatisfied with the new living place	*… Ms*. *X is very dissatisfied with the residence*. *She’s very bored … she says “not enough is going on” in the residence and that having a view over the parking lot isn’t very entertaining*. *She also complains about the fact that she can’t smoke in her apartment or have pets (Senior*, *74 years)*
	Caregiver’s feelings of guilt	*Ms*. *X feels a lot of guilt about the decisions that have been made for her loved one and finds the situation difficult (Daughter of a senior*, *65 years)*
	Feeling frustrated	*Mr*. *X is still frustrated with the situation (Spouse/partner of a senior*, *76 years)*
	Ambivalence	*Although she maintains that the decision is final*, *she still seems somewhat ambivalent (Senior*, *83 years)*
	Feeling of sadness	*Feels sad (Senior*, *85 years)*
		*Tears in her eyes when she talks about her mother moving somewhere else (Daughter of a senior*, *64 years)*
	Disagreement with family	*… She observes that making the decision to stay in her own place alienated her from her children*, *who didn’t agree with the decision*. *Is at ease with her choice and doesn’t regret anything (Senior*, *82 years)*
	Higher cost of living in residential setting	*Mr*. *X mentioned that his wife has moved into a private seniors’ residence … it’s very expensive in a private residence … He doesn’t know how he’s going to manage to pay for it all (Spouse/partner of a senior*, *73 years)*
	Higher cost of living at home and lack of public homecare services	*The participant hired a homecare worker who helps her every morning of the week*, *but her mother has to pay for it because she can’t get help from the [public home care services]*. *She was told that her mother should also have help getting ready for bed*, *but that would also be at their own expense (Daughter of a senior*, *59 years)*
	Lack of support for caregiver in taking care of the senior	*She herself has to make sure her mother’s okay every day*, *she can’t rely on help from the family or from social services (Daughter of a senior*, *59 years)*.
**Increased services**	Increased services	*Her mother’s functional abilities have deteriorated a lot but she wants to stay in her own place*. *The participant therefore organized for her to have additional services (Daughter of a senior*, *59 years)*
**Delay of relocation**	Long waiting time for relocation	*Ms*. *X mentions that there was a very long waiting time before she could move (Daughter of a senior*, *52 years)*

^a^Logbooks were completed by research assistants. A total of 79 logbooks were analyzed.

Negative outcomes included seniors’ dissatisfaction with the decision to move and with their new home, feelings of sadness, caregivers’ feelings of guilt, frustration, ambivalence, and higher costs of living or costs of health services:

*… his wife has moved into a private seniors’ residence … it’s very expensive in a private residence … He doesn’t know how he’s going to manage to pay for it all* (Spouse/partner of a senior, 73 years).

## Discussion

These results shed important light on the experiences of seniors and informal caregivers in decision-making about housing. In many cases there was a mismatch between the actual choice and their initial preference. For many seniors and caregivers, the decision-making process was reactive rather than considered, e.g. triggered by illness or a hospitalization that forced them to make hurried decisions without enough time to find out about their options. Caregivers of cognitively impaired seniors experienced decisional conflict and often lacked proper decisional support from healthcare professionals. While some decision experiences and outcomes were positive, most seniors and caregivers reported dissatisfaction, frustration, guilt, sadness and higher costs associated with new living arrangements. Our findings lead us to make the following observations.

First, caregivers and seniors frequently assumed an active role in housing decisions and yet in many instances their preference did not match the actual choice (more than half of those who moved would have preferred not to). Other studies have shown that housing decisions that did not match preferences may have been a result of poor decision support [40]. They may also have been driven by other considerations, such as serious health and safety concerns, problems with current living arrangements, and caregiver burden. These considerations are conceptualized in Wackerbarth’s Dynamic Model of the Caregiving Decision Process which distinguishes three main components in caregivers’ decision-making: the tolerance line, status points and decision events [41]. The tolerance line represents the upper boundary of what the caregiver perceives as a tolerable situation. Status points represent how well the caregiver is doing. As the status points approach the tolerance line, a decision to change should be made. The third component is the decision event. The caregiver typically makes a decision a) to make the caregiving more tolerable while avoiding or delaying a more drastic change or b) in reaction to a crisis [41]. Therefore, near or above the tolerance line and under conditions dictated by a crisis, the caregiver has to make a change to avoid a situation that could be dangerous for the caregiver, the care receiver, or both [41]. In these situations, despite clear preferences to remain at home, those making the decision saw no other option but relocation. These results highlight the importance of preparing for possible changes in advance to avoid having to make quick, reactive decisions [42].

Second, these results highlight that engaging seniors and family caregivers in discussions about future care when the elderly loved one is still able to participate is essential. This may be achieved by advance care planning (ACP). ACP is a process of reflection and communication in which someone who still has decision-making capacity makes decisions about future health and/or personal care options [43]. While it has been extensively studied for end-of-life decisions such as do-not-resuscitate orders [44], advance planning is important for housing decisions too, as they are often made *in extremis* without decision-making support, and often by caregivers as proxies for their loved ones [45]. Several studies have found that ACP is associated with positive results in a large range of end-of-life outcomes from health utilization (place of death, hospitalization, specific treatments) to economic outcomes (cost saving), and including patient/caregivers outcomes (satisfaction with care, concordance, physical or emotional distress) [44]. Moreover, ACP has the potential to promote patient autonomy and shared decision making and to improve quality of care at the end of life [46]. Home care teams can play an important role in this regard, helping seniors and their caregivers clarify the need for an eventual decision well before declining autonomy makes it urgent, presenting the options available with different scenarios as well as the pros and cons of each option, and initiating discussions about the preferences and values of seniors, caregivers, and other family members in all eventualities. An interprofessional shared decision-making approach is appropriate for this process [47], i.e. an approach that involves the whole health care team as well as the caregiver and senior, and the discussion should be routinely revisited as seniors’ situations and needs evolve. At times, the home care professionals may need to support the decision-making process by managing potential differences between the preferences of those making the decision for themselves or for their loved one and the real options available. When the preferences of seniors or caregivers cannot realistically be pursued, support for these individuals is needed to help them come to terms with the actual choices, as well as to cope with any disappointment or guilt that it may cause.

Third, these results highlight various difficulties encountered by caregivers making housing decisions for a loved one. Many experienced decisional conflict, and in some cases it was the heavy burden of caring for their loved one that prompted the housing decision and they felt guilty about the decision afterwards. Our recent systematic review on caregiver involvement in decision-making concluded that the extent to which health-related decisions are discussed with caregivers varies considerably and that indeed, caregivers have great difficulty contributing effectively and satisfactorily to decisions concerning their loved ones [20]. The difficulty of making a choice on behalf of someone else and of negotiating between one’s own preferences and those of a loved one can also explain caregivers’ discomfort about being involved in the decision, especially if that loved one has cognitive impairment [48]. In a Canadian survey of decision-making needs of adults faced with complex decisions, those making a decision about institutionalization of a family member were more likely to manifest decisional conflict than those making other types of decision [49]. Interactions with members of informal (e.g. family, friends, neighbours) and formal (e.g. non-regulated service providers) support networks can play a major role in the decision-making process regarding relocation of a cognitively impaired seniors [50], as confirmed by comments by caregivers in the qualitative analysis of logbooks. They mentioned that families may disagree with decisions made or put undue pressure on them. As reported in a previous study, this pressure can be exacerbated by factors such as the cost of the options, the short time in which they have to make the decision, and the perception that in reality they have few options to choose from (36, 37). The variety of caregivers’ experiences of the decision-making process may also be affected by the caregivers’ decision-making styles, which can be proactive, reactive or inactive [51]. Proactive caregivers collect information and plan ahead. They believe that being proactive will make the decision-making process easier as it will decrease the uncertainty of not knowing about alternatives and outcomes. Reactive caregivers make the decision only in reaction to an external trigger event. They try to prepare for decisions but become overwhelmed and give up, until an event forces them into action. Inactive caregivers are reluctant to take on the role of decision makers. They tend not to collect information or evaluate alternatives. They report that decisions were made by outside parties or by the disease itself [51].

Lastly, most studies on seniors with loss of autonomy that report factors predicting institutionalization are more interested in the association between seniors’, caregivers’ and system-level characteristics and the housing decision and less in the relationship between the decision-making process itself and the decision to move or not [52, 53]. This process itself with the various elements highlighted here, such as assumed role in decision-making and decisional conflict, could be equally strong predictors of the final decision. This study represents an interesting research avenue that may be pursued further.

This study has four limitations to consider. First, cognitively impaired seniors were considered to be unable to give consent based on the clinical judgement of their interprofessional home care team rather than based on validated measures. The perspectives of seniors who were cognitively impaired and yet still managed to participate in the housing decision-making process (i.e. because their caregivers spoke for them) may therefore have been missed. Second, the fact that the study samples were not dyads but two separate groups, i.e. cognitively competent seniors and caregivers of (other) cognitively impaired seniors, precluded meaningful comparison of these populations both from quantitative and qualitative perspectives. Instead, their respective perspectives were descriptively reported. Future studies should consider recruiting senior-caregiver dyads that would enable a more robust comparison of their respective perspectives and experiences regarding housing decisions. Third, those who volunteered to participate may have had better experiences of decision-making and been more likely to engage in it than those who could not be contacted or refused to participate. Therefore, these results might have overestimated the engagement of seniors and caregivers in the decision-making process. Finally, the qualitative analysis used observations made by RAs in the participant logbooks and was not collected directly from the seniors and caregivers themselves.

## Conclusion

This study is among the first to quantitatively report on decision-making among seniors and caregivers of cognitively impaired seniors regarding housing options. Our results suggest that, in the province of Quebec, Canada, seniors and caregivers have unmet needs for decision support, especially when their preferences do not match the actual choice. Advanced care planning regarding housing and better decision support are needed for these difficult decisions. Decision support interventions also need to address potential differences between what is preferred by the caregiver or their loved one, and what is either needed, or what is available. When the preferences of seniors or caregivers cannot realistically be pursued, they need support to help them come to terms with what is in fact the only option, as well as to cope with any disappointment or guilt that ensues. Furthermore, decision support interventions may need to be specifically tailored to caregivers given their difficult experiences of the decision-making process about their loved one’s housing options.

## Supporting information

S1 File Quantitative data for seniors. (XLSX)

S2 File Quantitative data for caregivers. (XLSX)

S3 File Logbook notes. (7Z)
